# Subgenual Cingulum Microstructure Supports Control of Emotional Conflict

**DOI:** 10.1093/cercor/bhw030

**Published:** 2016-04-05

**Authors:** Paul A. Keedwell, Amie N. Doidge, Marcel Meyer, Natalia Lawrence, Andrew D. Lawrence, Derek K. Jones

**Affiliations:** 1 MRC Centre for Psychiatric Genetics and Genomics; 2 Neuroscience and Mental Health Research Institute; 3 School of Psychology and Cardiff University Brain Research Imaging Centre (CUBRIC), Cardiff University, Cardiff, UK; 4 Centre for Affective Disorders, Institute of Psychiatry, London SE5 8AF, UK; 5 Experimental Psychology and Methods, Faculty of Psychology, Ruhr University Bochum, Bochum, Germany; 6 School of Psychology, University of Exeter, Devon, UK

**Keywords:** cingulum bundle, depression, diffusion MRI, emotional conflict, fractional anisotropy

## Abstract

Major depressive disorder (MDD) is associated with specific difficulties in attentional disengagement from negatively valenced material. Diffusion MRI studies have demonstrated altered white matter microstructure in the subgenual cingulum bundle (CB) in individuals with MDD, though the functional significance of these alterations has not been examined formally. This study explored whether individual differences in selective attention to negatively valenced stimuli are related to interindividual differences in subgenual CB microstructure. Forty-six individuals (21 with remitted MDD, 25 never depressed) completed an emotional Stroop task, using happy and angry distractor faces overlaid by pleasant or unpleasant target words and a control gender-based Stroop task. CBs were reconstructed in 38 individuals using diffusion-weighted imaging and tractography, and mean fractional anisotropy (FA) computed for the subgenual, retrosplenial, and parahippocampal subdivisions. No significant correlations were found between FA and performance in the control gender-based Stroop task in any CB region. However, the degree of interference produced by angry face distractors on time to identify pleasant words (emotional conflict) correlated selectively with FA in the subgenual CB (*r* = −0.53; *P* = 0.01). Higher FA was associated with reduced interference, irrespective of a diagnosis of MDD, suggesting that subgenual CB microstructure is functionally relevant for regulating attentional bias toward negative interpersonal stimuli.

## Introduction

Network models for mental illness propose that genetic and environmental risk factors produce alterations in corticolimbic circuit function that induce susceptibility to psychopathology (Drevets et al. 2008; Kim et al. 2011; Buckholtz and Meyer-Lindenberg 2012). In particular, risk for depression has been linked to altered functioning of a neural circuit encompassing the amygdala and regions of the ventral-rostral (pregenual–subgenual) anterior cingulate cortex (ACC) (Pezawas et al. 2005; Holmes et al. 2012).

The cingulum bundle (CB) is a major white matter pathway linking cortical regions implicated in cognitive control with limbic regions involved in emotion (Heilbronner and Haber 2014). It extends through frontal, parietal, and temporal cortices, connecting the cingulate cortex with proximal and distal cortical and subcortical structures. The CB consists of 3 subdivisions: subgenual, retrosplenial (or dorsal), and parahippocampal (or temporal) (Mufson and Pandya 1984). The subgenual CB links ventral and rostral ACC with the amygdala (Heilbronner and Haber 2014) and thus may be uniquely placed to play an integrative role in emotion–cognition interactions.

Previous diffusion MRI (dMRI) studies have found alterations in CB microstructure in individuals with, or at increased risk of, major depressive disorder (MDD). Using a targeted tractography-based approach, Cullen et al. (2010) found reduced fractional anisotropy (FA) (relative to matched controls) in the pathways between the ventral-rostral ACC and the right amygdala in adolescents with MDD. A further voxel-wise whole-brain analysis in the same cohort found reduced FA in parts of the supra-genual CB and adjacent uncinate fasciculi. Reduced FA in the anterior CB is also associated with increased risk (as indexed by positive family history) of MDD (Huang et al. 2011; Keedwell et al. 2012). Moreover, reduced FA in the anterior CB is linked to anhedonia (low positive affect) (Keedwell et al. 2012). Further supportive findings stem from a large-scale community-based sample, implicating decreased white matter volume in the anterior CB in preclinical depression (Vulser et al. 2015). This evidence converges to suggest that altered white matter microstructure in the CB could represent a trait marker of MDD. However, the functional significance of these alterations is unknown.

Convergent evidence suggests a key role for the ventral-rostral ACC in emotion regulation (Bush, Luu et al. 2000; Etkin and Schatzberg 2011; Etkin et al. 2011; Kim et al. 2011). In particular, it has been suggested that ventral-rostral ACC regions play a key role in monitoring and resolving “emotional conflict” arising from interference caused by task-irrelevant emotionally salient stimuli (Bishop et al. 2004). This parallels the role of the dorsal ACC in cognitive control (Botvinick et al. 2001). Several studies employing variants of an emotional Stroop task show that ventral-rostral ACC is activated during the processing of task-irrelevant negatively valenced emotional stimuli (Whalen et al. 1998; Bishop et al. 2004) of the kind that elicit enhanced negative attentional biases in individuals with mood disorder (Foland-Ross and Gotlib 2012). Compared with never depressed individuals, those with MDD are more distracted by negatively valenced stimuli, including angry and sad facial expressions, manifest in reduced accuracy and/or increased reaction times (Epp et al. 2012). The pattern in MDD represents an exaggerated form of the “negativity bias” (increased distraction from negative relative to positive stimuli) found in healthy young adults (Ebner and Johnson 2010). In addition, such distractibility becomes more marked with increasing severity of depression (Epp et al. 2012) and may potentially represent a cognitive vulnerability marker for MDD (Foland-Ross and Gotlib 2012). More recently, results from an emotional word–face Stroop paradigm, in which subjects categorize word stimuli according to their valence (positive, negative) while attempting to ignore emotionally congruent or incongruent distractor faces (Stenberg et al. 1998), suggest a role for ventral ACC in detecting and regulating emotional conflict (created by a word label incongruent with a facial expression) and in regulating amygdala response to distracting negatively valenced stimuli (Ochsner et al. 2009; Egner and Hirsch 2005; Etkin et al. 2006; Haas et al. 2006; Offringa et al. 2013). This task is also sensitive to the exaggerated negative attentional biases seen in depression (Hu et al. 2012).

Building on these findings of 1) altered CB microstructure in MDD; 2) ventral-rostral ACC involvement in emotional conflict processing; and 3) heightened emotional conflict from negative relative to positive emotional stimuli (especially in MDD), here we examine the relationship between the microstructure of distinct subdivisions of the CB and emotional conflict processing in never depressed (ND) and remitted depressed (RD) individuals using a word-face emotional Stroop task.

We hypothesized that (mirroring individuals with MDD) individuals with RD would demonstrate greater emotional Stroop conflict (i.e., slowing of reaction time) than ND controls when identifying pleasant words in the context of an angry face. Since increases in FA are associated, typically, with microstructural properties that are considered to support the efficient transfer of information along white matter (but see Tuch et al. 2005 and Jones, Knoesche et al. 2013 for discussion), we further hypothesized that these conflict or interference scores would correlate *negatively* with FA in the subgenual CB, such that higher FA would be related to reduced interference, in line with a role for the ventral-subgenual ACC and its interactions with the amygdala in detecting and regulating emotional distraction.

## Participants and Methods

### Participants

Forty-six healthy female participants aged 18–35 (mean age 22 years) were recruited from the staff and students of the School of Psychology, Cardiff University. Twenty-one individuals had a history of MDD (RD), while 25 had no history of MDD (ND). One participant had a history of bipolar mood disorder and was therefore excluded from further analyses. Due to technical problems during MRI data acquisition, we obtained dMRI data in *n* = 38 of the original sample (*n* = 17 RD individuals and *n* = 21 ND individuals), whereas behavioral data on emotional Stroop performance were collected in *n* = 46 individuals. From this (*n* = 46), the individual with a history of bipolar disorder was removed along with 2 individuals (1 ND and 1 RD) as a result of outlier removal procedures (see below), resulting in a final sample of *n* = 43 for the emotional Stroop paradigm. In the gender-based Stroop task (the control task, see below), 1 further ND individual was excluded (due to outlier procedures) (*N* = 43 and *N* = 42 therefore constituted the final sample sizes used for all behavioral analyses of the emotional and gender Stroop tasks, respectively). Both behavioral data and dMRI data were available for analysis in *n* = 35 (*n* = 17 RD and *n* = 18 ND) individuals for the emotional Stroop task and *n* = 34 for the gender Stroop task (*n* = 17 RD and *n* = 17 ND). All participants provided written informed consent. The study was approved by the School of Psychology Research Ethics Committee, Cardiff University.

Inclusion criteria included fluency in English, right-handedness, and normal vision. Exclusion criteria included a history of neurological disease, contra-indications to MRI, dyslexia, psychotropic medication and current depression, substance dependence, or other Axis I disorders, which were screened for using the Mini International Neuropsychiatric Inventory (MINI) (Sheehan et al. 1998). Past history of MDD in RD participants was confirmed using a modified version of the MINI (MINI-Plus version) (Bohnen et al. 2011) in conjunction with existing items for determining the number of previous episodes. This was also used to exclude ND participants if they had a personal history of depression. Remission was defined clinically using the MINI, which screens for depression symptoms in the previous 2 weeks. RD individuals had not taken medication during the previous 2 months. Participants also completed the National Adult Reading Test (NART; Nelson and Willison 1992), an estimate of verbal intelligence, the negative affect component of the Positive and Negative Affect Schedule (PANAS Negative) (Watson et al. 1988), the somatic anxiety component of the Anxiety Depression Distress Inventory-27 (ADDI SA; Osman et al. 2011) as well as the Beck Depression Inventory (BDI-II) (Beck et al. 1996) and other measures not relevant to the current investigation and that have been reported elsewhere (Bracht et al. 2015).

### Word-Face Stroop Task

We employed an emotional conflict task variant of the word-face Stroop paradigm developed in our lab (Meyer 2012). The word-face Stroop paradigm was selected, because it measures both semantic and response conflict between emotional cues (Etkin et al. 2006; Lin 2008).

**Figure 1.**
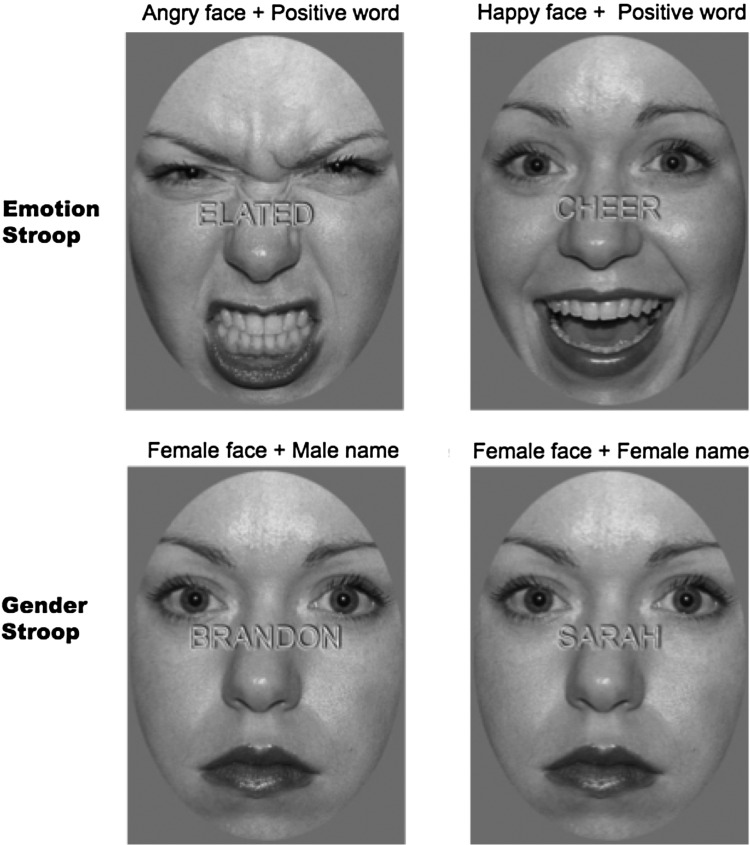
Examples of incongruent and congruent stimuli for the emotional and gender-based Stroop tasks.

All stimuli were prepared in Adobe Photoshop version 6 (Adobe Systems Inc., San Jose, CA, USA). We selected 9 Caucasian identities (4 females; Models no. 01, 02, 03, 06, 22, 23, 24, 25, 34), which ensured good legibility of the target words. Using the Psychophysics Toolbox extensions running in Matlab^®^ (MathWorks; Pelli 1997), distractor happy and angry faces were subtended at 10.96° × 7.84° visual angles (in height and width, respectively) and presented simultaneously with target words across the bridge of the nose (gray upper case letters in Arial subtending at maximum visual angles of 0.69° vertically and 6.49° horizontally). Examples are shown in Figure 1. The targets were happy-related words or angry-related words. Words were selected from the Affective Norms for English Words database (ANEW; Bradley et al. 1999), based on the categorical emotional ratings by Stevenson et al. (2007). They were matched for length, lexical complexity, and familiarity (Meyer 2012). They were further matched for emotional arousal using a published system (Stevenson et al. 2007; Meyer 2012). “Open-mouthed” angry faces and “exuberant” versions of the happy faces were selected from the NimStim database, because they are matched for levels of arousal (Tottenham et al. 2009). Happy and angry faces were chosen for 2 reasons: 1) their rater agreement, intensity ratings, and reaction times are similar (Palermo and Coltheart 2004) and 2) a sufficiently large number of well-matched words corresponding to these emotions can be identified (Stevenson et al. 2007). Furthermore, depression is linked to biased processing of both happy and angry faces, demonstrating increased sensitivity to angry faces (Leyman et al. 2007) and decreased sensitivity to happy faces (Joormann, Siemer, et al. 2007). Faces were cropped to an identical elliptical form to remove hair, shape, and background cues.

Participants were asked to identify whether words were either pleasant or unpleasant and to ignore the distractor faces. All trial types were presented with equal frequency in a randomized design. Based on the factorial combination of target word (emotional task: angry versus happy; gender task: male versus female) and congruency (congruent versus incongruent), there were 4 unique trial types for both the gender and emotion tasks. Distractor faces in the emotional task also varied as a function of gender. However, trials were collapsed in this respect, as this was not pertinent to the present investigation. Reaction times were corrected for accuracy (adjRT) (Townsend and Ashby 1983). Given our hypotheses, we were most concerned with conflict or interference scores for pleasant targets, that is, adjRTs when identifying pleasant words in the context of angry faces, minus adjRTs when identifying pleasant words in the context of happy faces (incongruent − congruent adjRTs, or emotional conflict).

As a control task, we used a “gender Stroop” task that comprised male or female facial stimuli with a calm expression (suitable as a neutral condition) from the same standard series and using the same identities, superimposed with congruent or incongruent gender-oriented names (based on UK census data on baby names). We selected calm faces as control stimuli for several reasons. Neutral facial expressions (an alternative stimulus set to calm faces) are commonly perceived as ambiguous in valence (Cooney et al. 2006), and their interpretation is easily rendered more negative through learned associations (Yoon and Zinbarg 2008) or individual differences (Donegan et al. 2003; Somerville et al. 2004). Past research has addressed this problem with artificially generated control stimuli, such as 25% happy faces (Phillips et al. 1997; Phillips et al. 2001; Mourao-Miranda et al. 2012). However, we opted for calm expressions for posing a perceptually similar, but emotionally less salient and thereby potentially “more neutral” alternative to neutral expressions (Tottenham et al. 2009). Piloting work by our research group indicated that calm expressions fared as well as neutral stimuli when measured on rating scales of arousal and valence, a conclusion that is further confirmed by prior work (Meyer 2012). Analogous to the emotion variant of the task, participants had to decide whether a target word was male or female and to ignore the distractor faces. Once again, words were matched for length, lexical complexity, and familiarity (Meyer 2012). A gender Stroop score was calculated in similar fashion to the emotional conflict measure described above (i.e., gender incongruent minus gender congruent adjRTs).

Trials began with the presentation of the fixation cross for 500 ms followed by the experimental stimuli (1000 ms). Participants' responses to the experimental stimuli were self-paced. Prior to the next trial, feedback in the form of the words “correct”/“incorrect” was displayed for 600 ms. The feedback was presented in white upper case letters in Courier New type, subtending at maximum visual angles of 0.57° vertically and 5.47° horizontally. Stimulus–response mapping was counterbalanced across participants (keys « c » and « v »), and every 144 trials participants were given the chance to take a break for as long as needed. For each Stroop task (i.e., emotion and gender), there were 2 practice blocks where participants were required to discriminate the target words. In the first practice block, only the word stimuli were displayed (to improve participants' accuracy in word discrimination), whereas the second practice block consisted of a shortened version of the experimental phase (48 trials per practice block) (Meyer 2012). Overall, there were 288 experimental trials for the emotional Stroop task versus 144 for the gender version—32 trials (40 trials) for each unique trial type with female (male) distractors.

Mean reaction time (RT) for correct responses and mean accuracy (ACC) data were computed. According to Ratcliff (1993), outlier removal based on SD is advisable in view of large variability of individual mean scores. The presence of outliers can lead to elevated error rates and substantial distortions of both parameter and statistic estimates when using either parametric or nonparametric tests (Osborne and Overbay 2004). We therefore adopted the following outlier procedure. Outlier removal was run separately for each group (RD vs. ND). For RT data, outliers were removed at the individual trial level (i.e., “raw” RT scores). Specifically, for any one participant, those individual RT scores that were >2.5 SDs from the participant's mean RT for a particular trial type were deleted along with their corresponding ACC values. For the emotion task, this resulted in a maximum of 5.6% of trials being excluded for a particular trial type (equal percentage for the RD and ND groups). For the gender task, in turn, a maximum of 7.5% of trials were removed for a particular trial type in the ND group (6.3% for the RD group). Mean RT was then divided by mean accuracy to create a new combined measure for every participant that linearly accounts for speed accuracy trade-offs (adjusted RT; adjRT) (Townsend and Ashby 1983). This was necessary since, for some trial types, error rates larger than 10% were found for a few participants. Subsequent analyses will therefore focus on this measure (adjRT). In a second step, outliers were removed for the adjRT data, that is, with regard to trial type across participants. In particular, for each trial type, we computed mean adjRTs across all participants of a particular group (RD vs. ND). Here, participants whose scores for one or more trial types were at least 2.5 SDs from the mean were excluded from the analyses. This step accounts for atypical factors affecting a participant’s performance (e.g., poor concentration). Based on this procedure, 1 participant was removed from each group of the emotion task (ND and RD). For the gender task, 1 RD and 2 ND individuals were excluded. One could argue that the 2-step outlier removal procedure, as used here, obscures group effects in that poor concentration might be an intrinsic feature of the subclinical profile of remitted depression. All data reported here were therefore reanalyzed without removal of reaction time outliers. Results showed the same pattern of effects.

### dMRI Procedure

Diffusion-weighted MR data were acquired on a 3 T GE HDx MRI system (General Electric Healthcare). Diffusion encoding gradients (*b* = 1200 s/mm^2^) were applied along 60 isotropically distributed directions (Jones et al. 1999). Six additional nondiffusion-weighted scans were collected as described in Bracht et al. (2015).

The data were corrected for participant motion and eddy current-induced distortions using an affine registration to the nondiffusion-weighted images, with appropriate reorienting of the encoding vectors (Leemans and Jones 2009). A single diffusion tensor model was fitted (Basser et al. 1994) to allow extraction of FA values. FA reflects the extent to which diffusion within biological tissue is anisotropic, or constrained along a single axis, and can range from 0 (fully isotropic) to 1 (fully anisotropic). The Damped Richardson-Lucy Algorithm (dRL) was used to estimate the fiber orientation density function (fODF) in each voxel (Tournier et al. 2004). Fiber tractography was carried out using *ExploreDTI* (version 4.8.1) (Leemans et al. 2009) following peaks in the fODF reconstructed from dRL (Jeurissen et al. 2011). For each voxel in the data set, streamlines were initiated along any peak in the fODF that exceeded an amplitude of 0.1 (thus, multiple fiber pathways could be generated from any voxel). Each streamline continued, in 0.5 mm steps, following the peak in the ODF that subtended the smallest angle to the incoming trajectory (subject to a threshold of 60° and an fODF amplitude threshold of 0.1).

**Figure 2.**
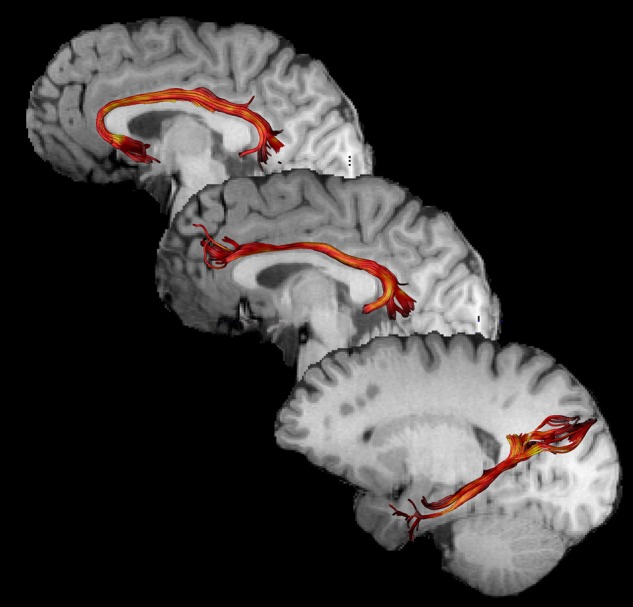
Examples of cingulum subdivision reconstructions. From top to bottom: subgenual, retrosplenial, and parahippocampal sections.

Once whole-brain tractography was complete, regions of interest (ROIs) were used to “virtually dissect” the 3 subsections of the CB—subgenual, retrosplenial (or dorsal), and parahippocampal (or temporal; Fig. 2). As described previously (Jones, Christiansen, et al. 2013), the FA map from each participant was registered to the FMRIB58 FA template in MNI space provided as part of the FSL software package (http://fsl.fmrib.ox.ac.uk/fsl/fslwiki/). The inverse of this spatial transformation was computed and subsequently used to translate a set of standardized waypoints, for virtual dissection of the CB and its subdivisions, to the native space of each subject. For each reconstruction thus obtained, the mean FA was calculated by averaging the FA values sampled at 0.5 mm steps along the entire length of each reconstructed tract or subsection (Jones et al. 2005).

### Statistical Analyses

For both the emotion and gender Stroop tasks, repeated-measures ANOVAs were used to determine main effects of target word, congruence with target word (congruent vs. incongruent faces), group (RD vs. ND), and their interactions. For all ANOVAs, planned comparisons were adjusted using Bonferroni correction. Student's *t*-tests were used to test group differences in FA and in individual difference variables (BDI, PANAS Negative, ADDI SA, age, and verbal IQ) and to examine group differences in Stroop interference scores (ΔadjRTs, see below). Pearson correlation coefficients (along with 95% confidence intervals, based on 5000 bootstrap samples) were used to explore associations between emotional and gender Stroop interference (conflict) scores and CB ROI FA (Bonferroni corrected). Associations were also examined between these measures (i.e., Stroop conflict and CB ROI FA) and the BDI/ADDI SA. Here, Bonferroni correction was applied separately for the following sets of correlations: 1) Stroop conflict versus BDI; 2) Stroop conflict versus ADDI SA; 3) CB ROI FA versus BDI; and 4) CB ROI FA versus the ADDI SA. Steiger Z tests were run to examine the difference between correlation coefficients for dependent samples (Steiger 1980). Steiger tests were performed using the spreadsheet available at: http://imaging.mrc-cbu.cam.ac.uk/statswiki/FAQ/WilliamsSPSS?action = AttachFile&do = view&target = steiger.xls. Default Bayesian linear regression analyses (Rouder and Morey 2012) were then performed to test the effect of all 3 FA CB subdivisions on emotional Stroop conflict scores (i.e., dependent variables: HA-HH ΔadjRTs and AH-AA ΔadjRTs).

#### Supplementary Bayesian Analysis

For our primary hypotheses, we also performed complementary Bayesian analyses. The Bayes factor (BF) indicates the relative strength of evidence for 2 alternative theories. A Bayes Factor score of *N*, comparing the alternative hypothesis to the null hypothesis (BF_10_) means that the data are *N* times more likely under the alternative than the null. BF_10_ values much >1 allow us to conclude that there is strong evidence for the alternative over the null. Conversely, BF_10_ values substantially lower than 1 provide strong evidence in favor of the null over the alternative. For example, a BF_10_ of 0.2 shows that the data are 5 times more likely under the null hypothesis than under the alternative. Default Bayes Factors, using a *β* prior of 1 for correlations, a Cauchy prior of 0.5 for regression analyses, and a Cauchy prior of 0.707 for *t*-tests (Rouder and Morey 2012; Wetzels and Wagenmakers 2012) were calculated using JASP (https://jasp-stats.org).

## Results

### Participant Characteristics

Participant characteristics are summarized in Table 1. RD and ND groups were well matched for age and estimated verbal IQ; the groups were still matched on these parameters for the final sample (*N* = 34) used for the analyses pertaining to FA in CB. ND participants had lower levels of depression and negative affect (as indexed by the BDI and PANAS Negative) than RD participants—no group differences in anxiety (ADDI SA) were observed. Of the RD individuals, 51% had experienced 1 episode, 5% 2, and 24% 3 episodes (mean = 2.81 episodes, SD = 3.25; range 1–15 episodes).

**Table 1:** Participant characteristics as a function of group (remitted depressed vs. never depressed)

	Remitted depressed: Mean (SD)	Never depressed: Mean (SD)	*t* value	Degrees of freedom	*P* value (2-sided)	95% Confidence interval of the difference		Hedges’ *g_s_*
						Lower	Upper	
Age	22.10 (3.46)	21.91 (3.58)	−0.17	42	0.87	−2.33	1.96	−0.05
Premorbid IQ	113.24 (4.27)	112.13 (4.66)	−0.82	42	0.42	−3.84	1.62	−0.24
BDI	11.29 (10.04)	2.61 (3.80)	−3.86	42	<0.01	−13.22	−4.14	−1.14
ADDI SA	12.43 (4.21)	11.04 (5.36)	−0.95	42	0.35	−4.34	1.57	0.27
PANAS negative	20.71 (7.42)	15.39 (4.02)	−3.00	42	0.01	−8.91	−1.74	0.89

Note: BDI, Beck Depression Inventory; ADDI SA, somatic anxiety component of the Anxiety Depression Distress Inventory; PANAS negative, negative affect component of the Positive and Negative Affect Schedule.

### Word-Face Stroop Results

#### Between Group Comparisons of Stroop Effects

**Figure 3.**
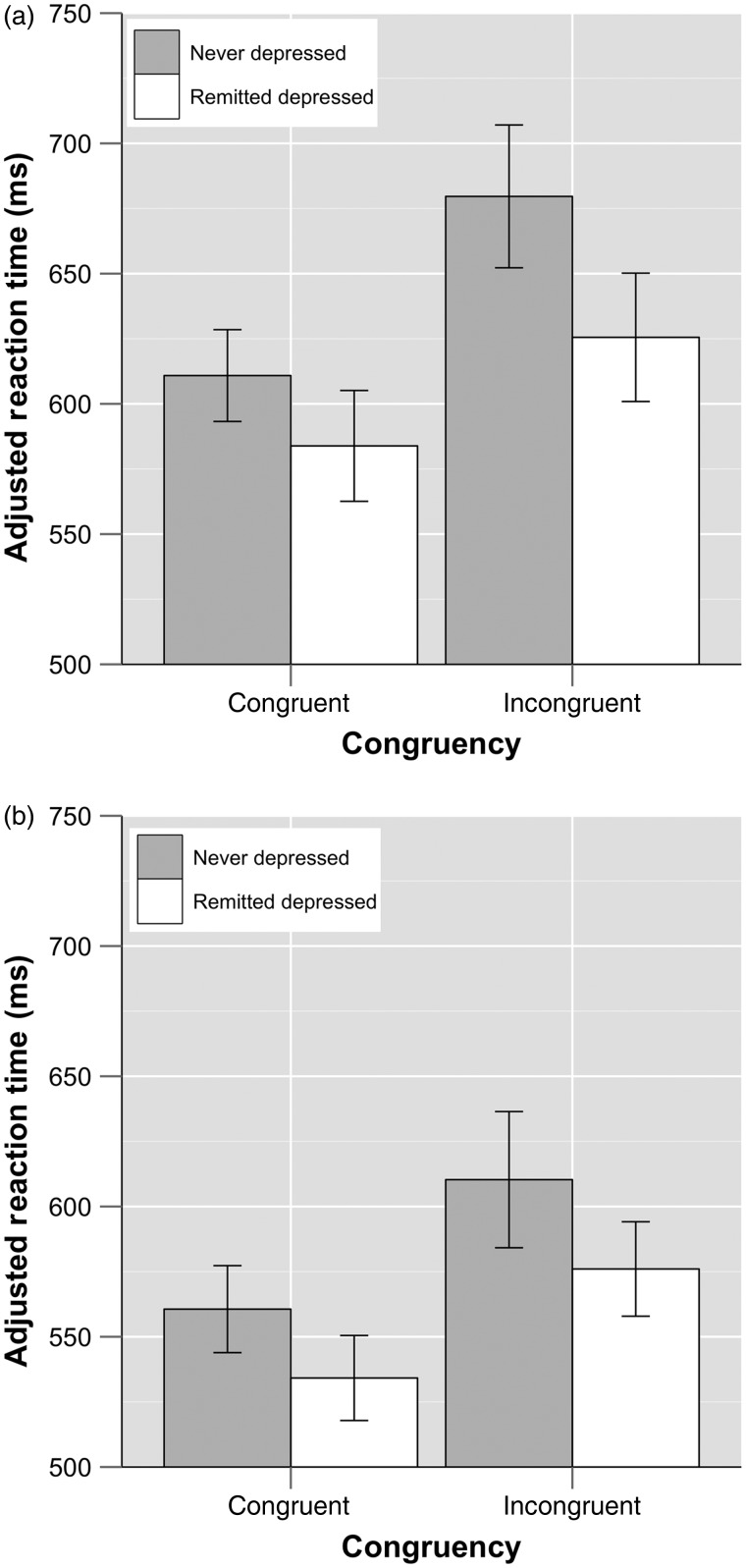
Effects of congruency × group in the (*a*) emotional and (*b*) gender Stroop tasks (with 95% confidence intervals).

For the emotional Stroop task, there was a significant congruency × group interaction (*F*_1, 41_ = 5.86, *P* = 0.02, *η*^2^ = 0.13). When this interaction was plotted (see Fig. 3), it was apparent that RD individuals performed better on incongruent word-face trials than ND individuals (*t*_(41)_ = 2.18, *P* = 0.04, 95% CI 4.03; 104.21, Hedges' *g_s_*= 0.66), with no significant difference emerging between these groups for congruent trials (*t*_(41)_ = 1.51, *P* = 0.14, 95% CI −9.02; 63.01, Hedges' *g_s_* = 0.45). Moreover, both groups responded more slowly to incongruent trials (ND: *t*_(22)_ = −7.35, *P* < 0.01, 95% CI −88.21; −49.38, Hedges' *g_av_*=0.95; RD: *t*_(19)_ = −7.85, *P* < 0.01, 95% CI −52.79; −30.56, Hedges' *g_av_* = 0.61), with an independent samples *t*-test indicating greater Stroop interference for ND relative to RD individuals (*t*_(41)_ = 2.42, *P* = 0.02, 95% CI 4.49; 40.75, Hedges' *g_s_* = 0.73). No group effects were obtained for the gender task (*P* > 0.05).

Stroop interference (conflict) scores were calculated for each target condition by subtracting performance on congruent trials from that of incongruent trials. This yielded the following conflict scores: 1) Emotional conflict: AH-AA ΔadjRTs (i.e., adjRT on incongruent trials for angry targets minus congruent ones) and HA-HH ΔadjRTs (i.e., adjRT on incongruent trials for happy targets minus congruent ones); 2) Gender conflict: MF-MM ΔadjRTs (i.e., adjRT on incongruent trials for male targets minus congruent ones) and FM-FF ΔadjRTs (i.e., adjRT on incongruent trials for female targets minus congruent ones). ΔadjRTs were not significantly different in RD versus ND individuals, including HA-HH ΔadjRTs (see Table 2).

**Table 2:** Stroop conflict measures as a function of group

	Remitted depressed: Mean (SD)	Never depressed: Mean (SD)	*t* value	Degrees of freedom	*P* value (1-tailed)	95% Confidence interval of the difference		Hedges’ *g_s_*	BF_-0_
						Lower	Upper		
HA-HH									
ΔadjRTs	56.04 (57.40)	84.99 (57.68)	1.65	41	0.95	−∞	58.56	0.49	0.13
AH-AA									
ΔadjRTs	27.31 (46.62)	52.60 (72.90)	1.33	41	0.91	−∞	57.26	0.40	0.15
MF-MM									
ΔadjRTs	15.00 (39.47)	25.01 (74.49)	0.54	40	0.70	−∞	41.45	0.16	0.22
FM-FF									
ΔadjRTs	68.70 (45.42)	74.42 (79.61)	0.28	40	0.61	−∞	39.86	0.09	0.25

Note: BF_-0_ = Bayes Factor testing in favor of the directional hypothesis that Stroop conflict is greater in remitted depressed individuals. The lower confidence bounds tend towards infinity due to the directional hypothesis.

#### Effects of Target Word, Congruency, and Their Interactions in the Whole Sample

**Figure 4.**
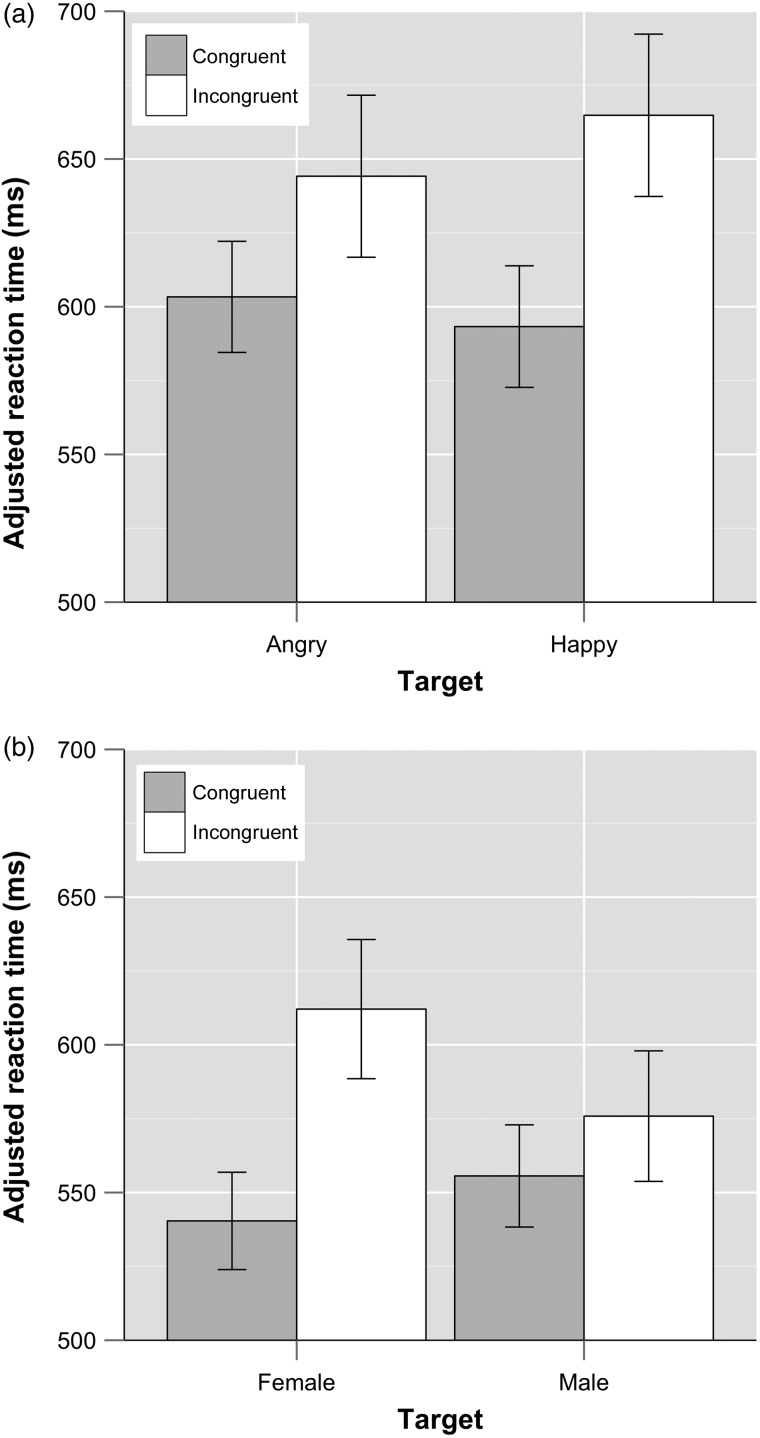
Mean adjusted reaction times for (*a*) the emotional and (*b*) the gender Stroop task with 95% confidence intervals. Trial types for (*a*): angry target word with angry distractor face, angry target word with happy distractor face, happy target word with happy distractor face, and happy target word with angry distractor face. Trial types for (*b*): female target word with female distractor face, female target word with male distractor face, male target word with male distractor face, and male target word with female distractor face (both lists from left).

Reaction times for emotion and gender Stroop tasks are shown in Figure 4. For both emotional and gender Stroop tasks*,* there was a significant effect of congruency (incongruent trials slower; emotional task: *F*_1, 41_ = 97.21, *P* < 0.01, *η*^2^ = 0.70; gender task: *F*_1, 40_ = 37.93, *P* < 0.01, *η*^2^ = 0.49). There was a significant main effect of target word for the gender task (male targets faster, *F*_1, 40_ = 8.03, *P* < 0.01, *η*^2^ = 0.17), but no main effect of target word for the emotional task (*F*_1, 41_ = 1.98, *P* = 0.17, *η*^2^ = 0.05). There was a significant target × congruency interaction for both the emotional task (*F*_1, 41_ = 4.46, *P* = 0.04, *η*^2^ = 0.10) and the gender task (*F*_1, 40_ = 16.73, *P* < 0.01, *η*^2^ = 0.30).

For the emotional task, planned comparisons for this interaction showed that on incongruent trials, performance was slower with happy (vs. angry) target words (*t* = 2.41, df = 42, *P* = 0.02, 95% CI 3.37; 37.88, Hedges' *g_av_* = 0.23). In contrast, on congruent trials, performance did not differ as a function of target word (*t* = 1.35, df = 42, *P* = 0.19, 95% CI −4.95; 25.08, Hedges' *g_av_* = 0.16). Also, for both target types, incongruent adjRTs were slower than congruent adjRTs (angry targets: *t* = −4.27, df = 42, *P* < 0.01, 95% CI −60.13; −21.54, Hedges' *g_av_* = 0.54; happy targets: *t* = 7.99, df = 42, *P* < 0.01, 95% CI 53.46; 89.59, Hedges' *g_av_* = 0.91). However, for happy targets, this interference effect was more prominent (paired *t*-test: *t* = −2.15, df = 42, *P* = 0.04, 95% CI −59.49; −1.89, Hedges' *g_rm_* = 0.50).

For the gender task, in turn, planned comparisons also revealed that performance on incongruent (vs. congruent) trials was significantly slower for both target types (male targets: *t* = −2.19, df = 41, *P* = 0.04, 95% CI −38.91; −1.57, Hedges' *g_av_* = 0.32; female targets: *t* = −7.16, df = 41, *P* < 0.01, 95% CI −91.92; −51.48, Hedges' *g_av_* = 1.11), with adjRTs for female targets yielding a greater difference in terms of Stroop interference (*t* = −4.14, df = 41, *P* < 0.01, 95% CI −76.58; −26.33, Hedges' *g_rm_* = 0.82). Additionally, adjRTs were slower for trials with male, as opposed to female, distractors (congruent trials: *t* = 2.20, df = 41, *P* = 0.04, 95% CI 1.24; 29.21, Hedges' *g_av_* = 0.28; incongruent trials: *t* = −4.75, df = 41, *P* < 0.01, 95% CI −51.64; −20.82, Hedges' *g_av_* = 0.49).

### dMRI Results

#### Group Comparisons of CB FA

There were no main effects of group on FA across the cingulum subdivisions (subgenual: *t*_(36)_ = 0.48, *P* = 0.64, 95% CI −0.02; 0.02, Hedges' *g*_s_ = 0.15; retrosplenial: *t*_(36)_ = −0.20, *P* = 0.84, 95% CI −0.20; 0.02, Hedges' *g*_s_ = −0.07; parahippocampal: *t*_(36)_ = 1.48, *P* = 0.15, 95% CI −0.04; 0.01, Hedges' *g*_s_ = 0.47).

#### Correlations Between Stroop Conflict Scores and CB FA

**Figure 5.**
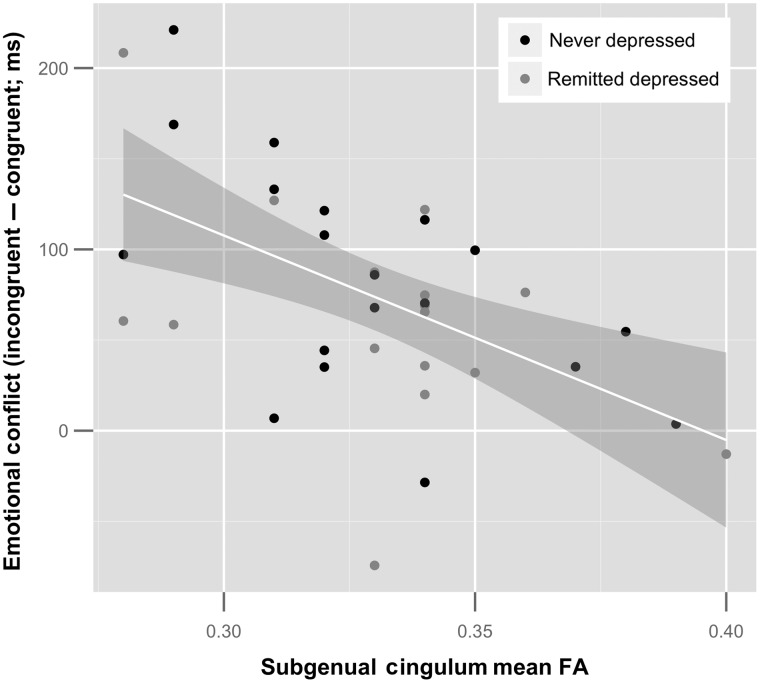
Negative correlation between the HA-HH ΔadjRT scores and mean FA in the subgenual cingulum bundles across the entire sample (*n* = 34, *r* = −0.53, *P* = 0.01, 95% CI −0.73; −0.26).

Given that conflict scores and CB FA did not differ by group, we examined the relation between these 2 variables across the whole sample. According to our key hypothesis, FA of the subgenual CB should be negatively associated with HA-HH ΔadjRTs in particular (i.e., individual differences in adjRT slowing created by a happy word label incongruent with an angry face) (Leyman et al. 2007; Ebner and Johnson 2010; Epp et al. 2012; Foland-Ross et al. 2012). For the emotional Stroop, HA-HH ΔadjRTs correlated significantly and negatively with FA in the subgenual CB (*r*_(34)_ = −0.53, *P* = 0.01, 95% CI −0.73; −0.26, BF_-0_ = 76), accounting for 28% of the variance (see Fig. 5), but not with other regions of the CB (see Table 3).

**Table 3:** Correlations between adjRT Stroop interference scores for gender and emotion tasks and mean FA values in the subdivisions of the cingulum bundle

Stroop conflict score	Cingulate region	*R*	*P*	95% CI (lower)	95% CI (upper)	*N*
HA-HH ΔadjRT	Parahippocampal	0.04	1.00	−0.20	0.34	34
	Retrosplenial	−0.26	1.00	−0.63	0.19	34
	Subgenual	**−0.53**	**0.01**	**−0.73**	**−0.26**	34
AH-AA ΔadjRT	Parahippocampal	−0.21	1.00	−0.52	0.22	34
	Retrosplenial	−0.01	1.00	−0.36	0.35	34
	Subgenual	0.05	1.00	−0.32	0.39	34
MF-MM ΔadjRT	Parahippocampal	0.10	1.00	−0.37	0.66	34
	Retrosplenial	0.00	1.00	−0.27	0.38	34
	Subgenual	0.07	1.00	−0.14	0.34	34
FM-FF ΔadjRT	Parahippocampal	−0.10	1.00	−0.42	0.29	34
	Retrosplenial	−0.24	1.00	−0.54	0.08	34
	Subgenual	−0.01	1.00	−0.28	0.25	34

Note: adjRT, mean adjusted reaction time; FA, fractional anisotropy for the subdivisions of the cingulum bundle. Bonferroni correction was applied to the p-values of Tables 3, 4 and 5 using the p.adjust function in R (http://www.R-project.org/).

Steiger Z tests confirmed that this correlation was specific to the subgenual CB. The correlation of mean HA-HH ΔadjRT with FA in the subgenual cingulum (*n* = 34) was significantly different from the correlation with FA in the parahippocampal cingulum (*z* = −2.68, ΔFisher zs = −0.63, SE = 0.24, *P* < 0.01, 1-tailed) as well as the retrosplenial cingulum (*z* = −1.58, ΔFisher zs = −0.32, SE = 0.20, *P* = 0.06, 1-tailed, marginally significant). Moreover, the correlation between FA in the subgenual cingulum and HA-HH ΔadjRTs differed significantly from the correlations with AH-AA ΔadjRTs (*z* = −2.28, ΔFisher zs = −0.640, SE = 0.28, *P* = 0.01, 1-tailed), MF-MM ΔadjRTs (*z* = −2.49, ΔFisher zs = −0.66, SE = 0.27, *P* < 0.01, 1-tailed) and FM-FF ΔadjRTs (*z* = −2.16, ΔFisher zs = −0.58, SE = 0.27, *P* = 0.02, 1-tailed).

No significant correlations were observed between AH-AA ΔadjRTs or the control gender Stroop conflict scores versus FA in any of the CB subdivisions (see Table 3), with default Bayesian tests for a correlation favoring the null hypothesis in all cases.

We also ran a default Bayesian linear regression analysis (Rouder and Morey 2012) with all 3 FA CB subdivisions as predictors and HA-HH ΔadjRTs as dependent variable. Results indicated that FA in the subgenual cingulum was the sole significant predictor in this model, that is, when holding the variance in the other CB subdivisions constant (subgenual cingulum: BF_10_ = 31; all other CB subdivisions BF_10_ < 1). A similar model with AH-AA ΔadjRTs as a dependent variable did not reach significance (BF_10_ < 1 for all predictors).

#### Effect of Subclinical Depression and Anxiety Symptoms on FA

There were no significant correlations between depression (BDI) or anxiety (ADDI SA) scores and FA in the CB subdivisions or between BDI/ADDI SA and conflict scores for emotion and gender tasks (see Tables 4 and 5).

**Table 4:** Correlations between Beck Depression Inventory (BDI) and ADDI Somatic Anxiety (ADDI SA) scores versus FA in cingulum bundles

Measure of negative affect	Cingulate region	*R*	*P*	95% CI (lower)	95% CI (upper)	*N*
BDI	Parahippocampal	−0.07	1.00	−0.33	0.21	37
	Retrosplenial	−0.10	1.00	−0.37	0.16	37
	Subgenual	0.14	1.00	−0.11	0.40	37
ADDI SA	Parahippocampal	0.03	1.00	−0.17	0.30	37
	Retrosplenial	0.16	1.00	−0.30	0.41	37
	Subgenual	0.27	0.31	−0.29	0.57	37

**Table 5:** Correlations between Beck Depression Inventory (BDI) and ADDI Somatic Anxiety (ADDI SA) scores versus adjRT difference scores for emotion and gender tasks

Measure of negative affect	Stroop conflict score	*R*	*P*	95% CI (lower)	95% CI (upper)	*N*
BDI	HA-HH ΔadjRT	−0.28	0.31	−0.47	−0.11	42
	AH-AA ΔadjRT	0.08	1.00	−0.15	0.34	42
	MF-MM ΔadjRT	−0.03	1.00	−0.25	0.20	42
	FM-FF ΔadjRT	0.18	1.00	−0.12	0.47	42
ADDI SA	HA-HH ΔadjRT	−0.29	0.25	−0.45	−0.15	42
	AH-AA ΔadjRT	−0.16	1.00	−0.35	0.13	42
	MF-MM ΔadjRT	0.11	1.00	−0.09	0.36	42
	FM-FF ΔadjRT	−0.01	1.00	−0.19	0.29	42

## Discussion

Ventral-rostral portions of the ACC have been implicated in the pathophysiology of mood disorders including depression and are thought to play a key role in emotion regulation, in part by controlling amygdala reactivity and selective attention to salient emotional stimuli (Etkin et al. 2011). The CB (specifically the subgenual portion) is the major association tract linking regions of the cingulate gyrus with both the amygdala and regions of prefrontal cortex (Heilbronner and Haber 2014), and so we set out to test the hypothesis that the subgenual CB plays a key role in regulating emotional conflict, to help understand the functional significance of white matter abnormalities previously reported in studies with patients with depression and related mood disorders.

An early influential functional parcellation of the cingulate cortex (Bush, Luu, et al. 2000) proposed a dissociation between a dorsal-caudal “cognitive” division and a rostral-ventral “affective” division. A more recent account argues that ventral-rostral portions of the ACC play a key role in emotion regulation (Etkin et al. 2011). In particular, research employing an emotional word-face Stroop task similar to the one used here has suggested that ventral-rostral ACC and related regions of medial PFC are involved in processing the conflict arising from emotionally salient but task-irrelevant input—a role analogous to that of dorsal ACC in cognitive conflict processing (Botvinick et al. 2001). Consistent with this suggestion, several functional imaging studies have observed activation of the ventral-rostral ACC and mPFC during various forms of emotional conflict or interference (Whalen et al. 1998; Bishop et al. 2004; Etkin et al. 2006; Hare et al. 2008; Etkin and Schatzberg 2011). This activity, which appears to regulate the degree of interference from emotional distractors (Egner et al. 2008), is altered in mood disorders (Etkin et al. 2011; Offringa et al. 2013) and in healthy individuals with high levels of trait negative emotionality (Bishop et al. 2004). Together these findings raise the question of whether microstructural variation in the anterior cingulum that connects ventral-rostral ACC and mPFC with regions including the amygdala (Heilbronner and Haber 2014) plays a critical role in mediating inter-individual differences in emotional conflict processing.

Our primary novel finding was that degree of emotional conflict (i.e., the slowing of reaction time in a word-face Stroop when labeling a happy word superimposed on an incongruent angry face, and presumably resulting from an inability to disengage attention from the angry face) correlated negatively with subgenual CB FA, but not with FA in the retrosplenial or parahippocampal CB subdivisions, such that higher subgenual CB FA was associated with lower levels of emotional conflict. Since increases in FA are associated, typically, with microstructural properties that are considered to support the efficient transfer of information along white matter (but see below), our findings do indeed suggest that some aspect of tissue microstructure (characterized by FA) of the subgenual CB plays a critical role in determining interindividual differences in emotional conflict processing, presumably by influencing efficiency of information transfer between the ACC and related structures.

In line with the proposed functional division of the dorsal and ventral ACC (Bush, Luu et al. 2000; Egner et al. 2008; Etkin et al. 2011), here emotional word-face Stroop task performance was not correlated with FA in more dorsal and caudal regions of the CB. Previous imaging studies including both cognitive and emotional conflict tasks (similar to the one used here) have shown that dorsal-caudal cingulate is activated by both cognitive (classical Stroop Interference) and emotional conflict, but that the ventral-rostral cingulate is selectively activated during emotional conflict (Whalen et al. 1998; Egner et al. 2008; Hare et al. 2008; Ochsner et al. 2009; Etkin et al. 2011). We have demonstrated, through the use of our gender control task, that subgenual cingulum FA is not a generic correlate of performance on Stroop tasks per se*.* In the 2 tasks explored here, subgenual CB FA correlated specifically with emotional, as opposed to “cold” cognitive conflict. In line with our findings, dual-task interference studies reveal that conflict regulation in emotional and nonemotional Stroop tasks are dissociable on a functional level (Soutschek and Schubert 2013). Our results thus confirm a unique role for ventral-rostral ACC networks in regulating emotional conflict.

The subgenual CB contains those fibers linking ventral-rostral ACC with the amygdala and other regions involved in emotion and face processing (Heilbronner and Haber 2014). Studies of functional connectivity with fMRI suggest that ventral-rostral ACC may operate at least in part by regulating activity within the amygdala (Mathews et al. 2004; Egner et al. 2008; Smith et al. 2012). Our findings extend this work by demonstrating a “structural realization” of this functional connectivity, showing that interindividual variation specifically in subgenual CB microstructure plays a key role in mediating differences in the ability to detect and regulate emotional conflict, presumably by modulating the efficiency of information processing in corticolimbic circuitry. In contrast, variation in microstructure in more dorsal cingulum white matter tracts devoid of amygdala fibers (Heilbronner and Haber 2014) is less critical to individual variation in emotional conflict processing.

The role of ventral ACC regions in regulating emotional conflict has been suggested to represent one aspect of a broader role in emotion regulation (Etkin et al. 2011). In particular, it has been argued that the regulation of emotional conflict involves circuitry that overlaps with fear extinction. It is notable then that a recent study found that individual variation in anterior CB microstructure also predicted individual variation in fear extinction (Fani et al. 2015). Together, these findings suggest a broader role for the subgenual CB in emotion regulation.

In contrast to Metzler-Baddeley et al. (2012), we did not find that variation in dorsal cingulum FA predicted individual variation in cognitive control (gender Stroop). The disparity in findings could represent differences in the Stroop task used (the previous study used the classic color-word Stroop) or the population (the individuals in Metzler-Baddeley et al.'s study were healthy older individuals and patients with mild cognitive impairment). Lesion evidence that the dorsal cingulum is critical for cognitive conflict processing is somewhat inconsistent (Ochsner et al. 2001; Fellows and Farah 2005) and variation in other regions critical to implementing cognitive control (e.g., dorsolateral prefrontal cortex) (Botvinick et al. 2001) may be an important source of individual differences in nonemotional Stroop performance.

As an aside, results in the gender Stroop task suggested that male distractors in particular impaired RT performance in our female sample. Research has shown an own-gender bias in face recognition, indicative of improved processing efficiency and enhanced typicality of female faces in women (Vokey and Read 1988; Wright and Sladden 2003; Cellerino et al. 2004; Loven et al. 2011). The comparative “atypicality” and processing inefficiency of male face recognition in women may then account to some degree for the detrimental performance on trials with male distractor faces in our sample of women. Other work has revealed that depression is more common in women than in men, which is partly reflective of gender differences in emotion regulation (Nolen-Hoeksema 2012). Moreover, FA in the CB has been found to be lower in females versus males (Johnson et al. 2013). Future studies may therefore benefit from the inclusion of both participant genders to examine potential gender differences in the relation between CB microstructure and (gender and emotion) conflict processing.

An exaggerated attentional bias toward negative facial emotions has been previously reported in acute depression (Gotlib et al. 2004; Foland-Ross and Gotlib 2012) and at-risk individuals (Joormann, Talbot, et al. 2007), and some studies have observed persistent emotional (Joormann and Gotlib 2007) and even nonemotional Stroop impairment in remitted depression (Trichard et al. 1995; Nakano et al. 2008). However, the effects are sensitive to demographic and illness factors and appear when subjects are induced into a negative mood (Joormann and Gotlib 2007). In this study, RD individuals did not demonstrate significantly increased emotional conflict from the negative face distractors (longer HA-HH adjRTs) compared with control participants (see also Etkin and Schatzberg 2011). The lack of a significant group difference in performance in our sample could reflect several factors. For example, it could reflect the positive remedial effects of previous treatment. Moreover, the majority of our sample had experienced only 1 episode of depression. Recent longitudinal evidence suggests that persistent cognitive impairment is observed in recurrent depression but not after single episodes (Maeshima et al. 2012). Recent research has also shown that higher general cognitive function is related to lower levels of cognitive bias (Hakamata et al. 2014) and, in turn, may have served as a protective factor in our RD sample (Pargas et al. 2010), thereby reducing potential group effects. Relatedly, the young age of our participants might be a factor in determining preserved performance, which might potentially reflect compensatory neural activity as recently demonstrated in an fMRI study of the emotional face-word Stroop (Etkin and Schatzberg 2011). Persisting executive cognitive deficits following remission, including classic Stroop performance, are more common in elderly depressed individuals (Nakano et al. 2008), and depression-related cognitive deficits increase with age in elderly patients (Butters et al. 2004; Bhalla et al. 2009).

Strikingly, we found that RD individuals showed improved performance on incongruent trials in the emotional Stroop task. Again, this could signify positive remedial effects. As noted by Harmer et al. (2009), emotional processing is often altered before direct effects on mood can be detected. This might explain why we found improvements in Stroop performance, whereas negative mood ratings were still (comparatively) elevated in the RD group, albeit at a nonclinical levels (see below). Recent research has also suggested that the degree of impaired cognitive control of attention to emotion in remitted depression prospectively predicts recurrence of depression (De Raedt et al. 2010; Joormann and D'Avanzato 2010; Demeyer et al. 2012). An alternative explanation could thus be that the current sample contains those individuals who were able to compensate for any such impairment and therefore were somewhat less likely to be suffering ongoing recurrent episodes, indicative of a potential selection bias.

RD individuals also did not exhibit reduced FA in the cingulum compared with ND individuals. Again, this might reflect specific characteristics of the sample population or positive effects of previous treatment. Nevertheless, our study clearly demonstrates that interindividual variation in the microstructure of white matter fiber tracts linking ventral ACC with the amygdala, regardless of depression status, is related to the ability to regulate attention to emotional stimuli (for a similar finding regarding functional connectivity between ventral ACC and amygdala, see (Etkin and Schatzberg 2011)).

Our study had some limitations. The RD group had some residual depressive symptoms as indicated by increased BDI scores. However, the mean score was subthreshold for clinical depression, and current depression had been excluded using structured interview. Furthermore, BDI scores did not correlate with our main outcome measures. We recruited only unmedicated RD individuals. This excludes the possibility that medication could be affecting emotional word-face Stroop performance at the time of testing or white matter microstructure independent of clinical status. However, the disadvantage of this approach is that the sample population is not representative of all RD individuals, many of whom would require medication to remain in remission. Further work is needed in individuals with unmedicated acute depression.

Due to our specific a priori anatomical focus on ACC-amygdala connectivity (Egner et al. 2008), we restricted our tractography analysis to the CB. Other white matter tracts, in particular the uncinate fasciculus (Heilbronner and Haber 2014), have also been implicated in the pathophysiology of mood disorders (Versace et al. 2015) and in attentional bias to threat (Carlson et al. 2014). Future studies, using novel MRI-based techniques (De Santis et al. 2015), will be required to more fully characterize the role of specific white matter tracts in emotional conflict processing.

Biological interpretation of interindividual differences in FA is challenging (Jones 2010; Jones, Knoesche, et al. 2013), since FA is influenced by a number of possible microstructural properties, including myelination, axonal diameter, and/or fiber density. Future work should use emerging methods to estimate axonal density (Assaf and Basser 2005; Assaf et al. 2008) and myelin water fraction (Deoni et al. 2008) in the cingulum and determine their relationship to emotional conflict processing.

This is the first study to establish a link between white matter microstructure in the subgenual cingulum and emotional conflict processing, as indexed by performance on the word-face emotional Stroop. Our results suggest that high FA in the subgenual CB is linked to the effective control of attentional capture by negative interpersonal stimuli and hence minimizing their subsequent interference on task performance. This finding provides new insights into why some individuals experience greater difficulties in implementing effective emotion regulation, a key skill for appropriate emotional and social functioning.

## Funding

P.A.K. was funded by the Higher Education Funding Council for Wales (HEFCW) and an Academy of Medical Sciences and Wellcome Trust Starter Grant (AJ17102004). M.M. received an EPSRC Doctoral Training Grant. This work was also supported by a Marie Curie fellowship to Marcel Meyer and received funding from the European Union Seventh Framework Programme (FP7/2007-2013) under grant agreement no. 267171. D.K.J. was funded by HEFCW and received grants from the MS Society, a Wellcome Trust New Investigator Award, a Wellcome Trust Multi User Equipment Grant and Medical Research Council, and Wellcome Trust project grants. A.N.D. was supported by the Wellcome Trust PhD schemes. N.L. was funded by HEFCW. A.D.L. was funded by HEFCW. He also received grants from the ESRC, Wellcome Trust, and NISCHR. Funding to pay the Open Access publication charges for this article was provided by The Wellcome Trust
